# Digital Leg Volume Quantification: Precision Assessment of a Novel Workflow Based on Single Capture Three-dimensional Whole-Body Surface Imaging

**DOI:** 10.1007/s10278-021-00493-8

**Published:** 2021-09-28

**Authors:** Lucas Etzel, Thilo L. Schenck, Riccardo E. Giunta, Zhouxiao Li, Ya Xu, Konstantin C. Koban

**Affiliations:** Division of Hand, Plastic and Aesthetic Surgery, University Hospital, LMU Munich, Pettenkoferstraße 8a, 80336 Munich, Germany

**Keywords:** 3D surface scanner, Leg volumetry, Device validation, Whole-body scan, Leg edema, Structured-light scanning

## Abstract

Whole-body three-dimensional surface imaging (3DSI) offers the ability to monitor morphologic changes in multiple areas without the need to individually scan every anatomical region of interest. One area of application is the digital quantification of leg volume. Certain types of morphology do not permit complete circumferential scan of the leg surface. A workflow capable of precisely estimating the missing data is therefore required. We thus aimed to describe and apply a novel workflow to collect bilateral leg volume measurements from whole-body 3D surface scans regardless of leg morphology and to assess workflow precision. For each study participant, whole-body 3DSI was conducted twice successively in a single session with subject repositioning between scans. Paired samples of bilateral leg volume were calculated from the 3D surface data, with workflow variations for complete and limited leg surface visibility. Workflow precision was assessed by calculating the relative percent differences between repeated leg volumes. A total of 82 subjects were included in this study. The mean relative differences between paired left and right leg volumes were 0.73 ± 0.62% and 0.82 ± 0.65%. The workflow variations for completely and partially visible leg surfaces yielded similarly low values. The workflow examined in this study provides a precise method to digitally monitor leg volume regardless of leg morphology. It could aid in objectively comparing medical treatment options of the leg in a clinical setting. Whole-body scans acquired using the described 3DSI routine may allow simultaneous assessment of other changes in body morphology after further validation.

## Introduction

Three-dimensional surface imaging (3DSI) is increasingly gaining recognition as a valuable tool for the objective documentation of volumetric changes in body morphology. The use of 3D scanning has recently been investigated regarding its usefulness for the documentation of body shape in bariatric surgery [1]. It is also being used for ever more complex tasks in maxillofacial surgery [2] and is finding application for augmented reality and virtual reality [3]. Clinicians of a growing number of medical disciplines effectively apply this radiation-free and cost-efficient technology to monitor specific anatomical regions, such as the breast [4, 5], face [6, 7], or leg [7, 8].

However, only those parts of the body that are visible during the scanning process can be mapped reliably. This is especially relevant when wanting to assess the lower extremity, as part of the surface of each leg may be obscured by the other leg [9].

A variety of investigations have been conducted to identify an objective, fast, cost-efficient, precise, and non-invasive method to measure leg volume. Presently used methods to quantify limb volume changes include standardized tape measurements to estimate volume, water displacement, perometry, computer tomography (CT), and magnetic resonance imaging (MRI). Numerous studies have compared these methods [8, 10–15]. While they are all routinely applied in clinical practice, each comes with certain benefits and limitations. Tape measurements have been shown to be of limited reliability [11]. Volume measurements through use of water displacement represent a cumbersome method that is unsuited for patients with open skin lesions of the leg [12, 13]. The use of MRI or CT offers the beneficial ability to monitor the sub-surface morphology. However, these methods will remain time-consuming or invasive and are thus less useful for routine monitoring of leg volume.

To find an objective and simple alternative to these methods, recent studies have examined the use of 3DSI for leg volume analyses [8, 9, 13, 16, 17]. Clinical application for the documentation of body morphology is associated with various benefits. The technology provides a contact- and radiation-free method to create digital surface maps. As such it is fast, cheap, and non-invasive when compared to CT and MRI. 3D scanning can be outsourced to trained personnel other than the treating physician. It requires less effort than traditional measurement techniques such as water displacement or when collecting large data samples using a tape measure or calipers.

By following a standardized whole-body acquisition process, the use of 3DSI supplies a digital copy of the visible surface. This can be analyzed to monitor the shape and size of the entire human body regarding distances, surface areas, and total body or segmental volumes [18–21]. This application of handheld 3D scanners can allow the assessor to monitor multiple areas of interest without the need to individually scan every anatomical region in question [19, 22]. It also offers the benefit of simultaneously documenting other anatomical areas which may prove to be of interest during a later clinical evaluation.

While various studies have examined the use of 3DSI to digitally assess leg morphology, it is common practice to either reposition each leg for individual scanning [8, 9, 17], or to scan one pose with a defined inter-foot distance [23]. Depending on leg morphology, such a technique may however still fail to allow visibility of the entire leg circumference in subjects with high leg volume. This creates the need for a standardized pose that allows as much leg visibility as possible without repositioning and re-scanning multiple areas. The concept of gathering leg volume measurements from whole-body 3DSI has been addressed in several studies [24, 25]. However, there are types of body morphology that do not allow for full leg visibility with this technique. In such cases the use of an estimation process is necessary to fill holes in the 3D leg surface data.

Our solution included the development of a workflow to acquire a complete set of whole-body 3D surface data during a single scanning procedure, i.e., without merging individual 3D scans of parts of the body. These data were then used to precisely quantify bilateral leg volume regardless of leg shape and volume, while allowing further analysis of other anatomical regions of interest. To cope with varying leg morphology, two workflow variations were devised. For subjects in which the entire circumference of each leg was visible in the standard pose, we applied the complete leg visibility workflow variation. In cases where leg morphology caused insufficient exposure of the leg surface, a limited leg visibility workflow variation was applied to estimate the missing surface data and thus still enable precise leg volume quantification. With these workflow variations, we were able to compare leg volumes for subjects with a wide range of leg morphologies.

In this study, the authors wanted to investigate the usefulness of a 3DSI-based workflow for the objective documentation of leg volume. The aim of this study was to describe and apply a novel workflow and to assess workflow precision while comparing two workflow variations. The workflow variations were compared to assess whether the method can precisely gauge leg volume regardless of leg morphology. Our objectives were to collect two repeated samples of bilateral leg volumes from all subjects included in a suitable study cohort and to calculate the respective mean volumes as well as the absolute and relative percent differences between these paired samples of volumetric data.

## Patients, Materials, and Methods

### Study Population

Patients over the age of 18 years who presented themselves for medical consultation at our hospital department between July 2017 and January 2020 were approached for study enrollment. The subjects were recruited from a sample of patients receiving body-contouring surgery, patients with lipedema, as well as other plastic surgical patients without leg issues, e.g., breast cancer patients. We aimed to recruit a cohort with a wide body-mass-index (BMI) range, and to include subjects with diverse leg morphology and varying degrees of leg volume. Volunteers were excluded if presenting with orthopedic or neurological conditions likely to intefere with the imaging procedure.

### Data Collection and Workflow Protocol

Two sets of whole-body 3D surface data were acquired for each subject. After a first 3D surface scan, subjects were allowed to move and were then repositioned in the same pose for the second scan. We were thus able to examine the reproducibility of measurements.

By adhering to the following standardized imaging procedure and two variations of a novel leg volume analysis workflow, paired bilateral leg volume measurements were recorded for each participant. Prior to 3DSI, the respective imaging operator briefed the subject on the imaging procedure, obtained written informed consent, and recorded data regarding age, sex, and BMI.

The handheld and wireless structured-light Thor3D Scanner (Thor3D, Moscow, Russia) was used together with an automated turntable (Thor3D, Moscow, Russia) as a portable consumer grade whole-body imaging system. Technical details as specified by the manufacturer included a scanning accuracy of up to 0.2 mm and a resolution of up to 1 mm with a frame rate of up to 10 per second. The motorized turntable was capable of a rotational speed of 0.25–6.5 rpm. It was manually marked with areas for standardized foot positioning at an inter-foot distance of about 40 cm.

To ensure high scan quality, the subjects were requested to remove all jewelry and to tie back long hair as well as to remove all clothing apart from tight fitting undergarments. 3DSI was conducted with the subjects standing freely in an upright position with arms spread and the hands resting on the hips. The subjects were instructed to breathe freely for the duration of 3DSI, while trying to alter their body pose as little as possible. Subjects were requested to keep their eyes open, as preliminary tests showed that closed eyes increased the amount of swaying motion. At a scanning distance of about 1 m, the imaging operator performed a systematic scan sequence by moving the imaging focus from the feet toward the head. While taking advantage of the turntable rotation to circumferentially map all visible surface areas of the body, each set of whole-body 3D surface data was acquired in a single scanning procedure. The mean scan duration was about 45 s. The resulting raw data were transferred via USB flash drive to a commercial desktop computer running Microsoft Windows 10 for data processing.

**Fig. 1:**
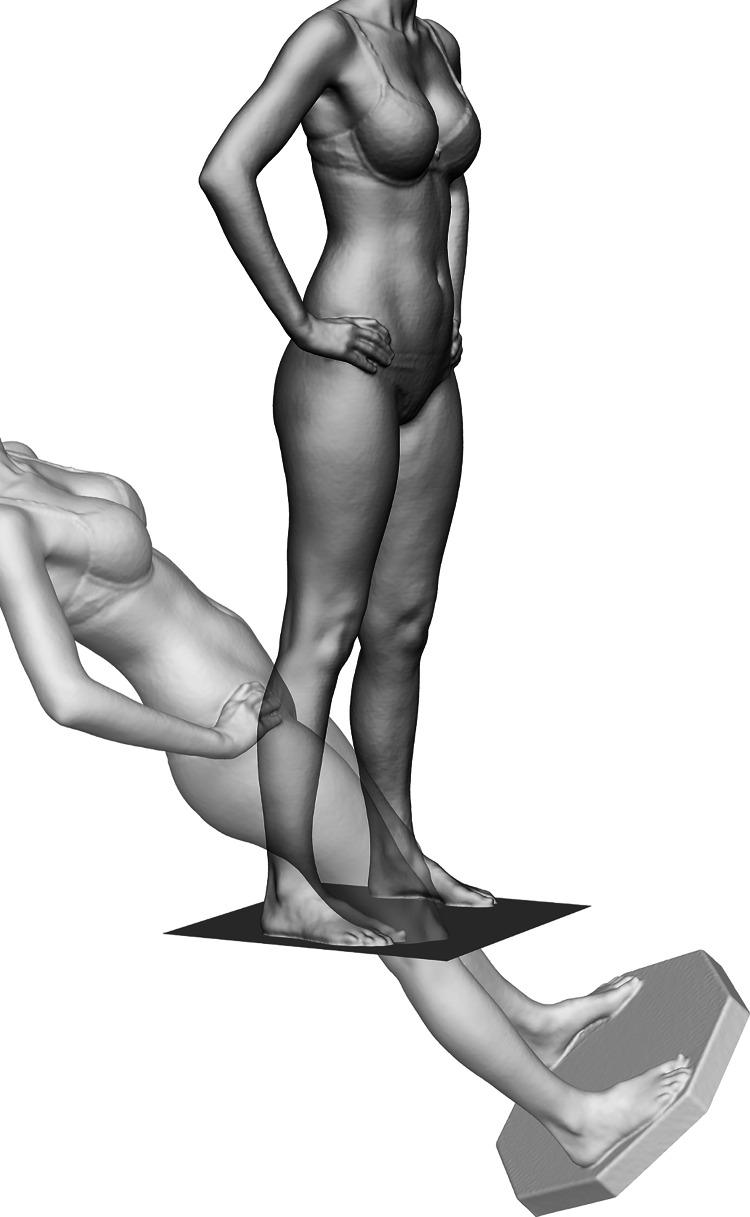
Orientation of a whole-body scan by aligning the flat turntable surface with a predefined transverse plane object at the coordinate system origin

Using predetermined settings based on those recommended by the manufacturer for whole-body scanning, data were processed using the Thor3D Windows desktop software and subsequently exported into the Geomagic 2014 software (3D Systems, Rock Hill, SC, USA). To allow for comparison, each resulting set of whole-body 3D surface data needed to be rotated to a standard orientation. To this purpose, the visible area of the flat turntable surface from each scan was aligned with a predefined transverse plane object at the coordinate system origin. The whole-body 3D surface data were then axially rotated to correctly face the frontal plane. The alignment process of the whole-body 3D surface data is depicted in Fig. 1.

**Fig. 2:**
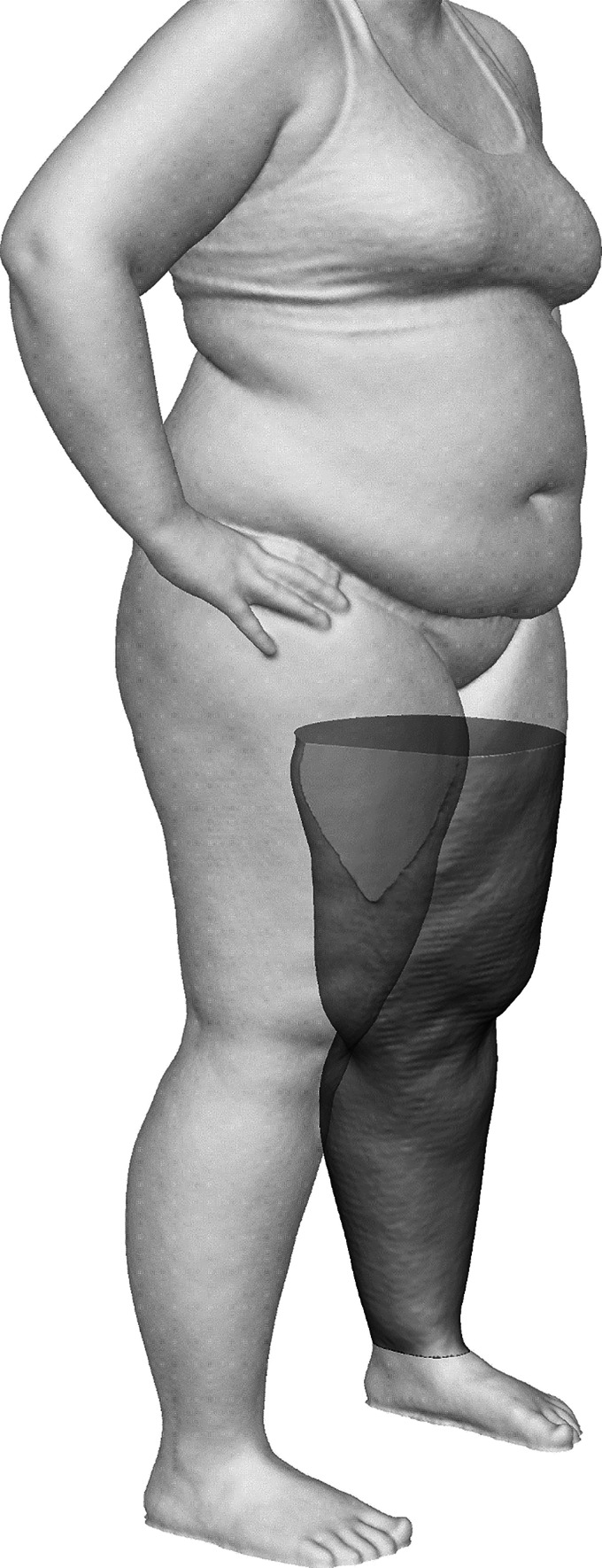
Exemplary illustration of the left leg segment of a whole-body scan processed using the limited leg visibility workflow variation

Leg segments were obtained for volumetric analysis by cropping the 3D surface data above and below transverse planes at the height of the infragluteal fold and the lateral malleolus, respectively. The transverse planes were set at a specific height in millimeters over the point of origin as defined by the turntable surface. These heights were then used for each leg of both sets of whole-body 3D surface scans. Thus, we were able to reproducibly crop the leg segments for volumetric analysis within the 3D surface data of the same subject. In cases where the two resulting legs segments were completely separate, the 3D surface data were submitted to the complete leg visibility workflow. In cases where the cropped segments showed fusing of the legs in the medial thigh area, the 3D surface data were submitted to the limited leg visibility workflow. This included an extra step of data processing, in which the computed 3D surface data were separated in the median sagittal plane between the fused legs. To estimate the medial boundaries of each respective leg, the missing 3D surface data were then interpolated by applying the flat plane hole-filling algorithm within the Geomagic 2014 software. An example of a leg segment from whole-body 3D surface data processed using the limited leg visibility workflow variation is highlighted in Fig. 2. Subsequently, the mesh doctor function within the Geomagic 2014 software was applied for scan cleanup by means of spike-edge removal and small hole filling, before saving each separated leg into individual.stl files.

The final leg segments were exported into the Mirror medical imaging software (Canfield Scientific Inc, Parsippany, NJ, USA). The in-software volume measurement function was used to quantify the leg segments. The output in the Mirror analysis log was saved in a .csv file ready for data transformation.

### Study Data Analysis

Data transformation and analysis, as well as scientific writing and table and graph creation, was conducted using the R software environment for statistical computing and graphics (version 4.0.0) [27] with RStudio (Version 1.3.1093) [28], R Markdown [29], and selected R packages [30–33]. A *p* value < 0.05 was considered statistically significant. Sample normality was analyzed using *q*-*q* plots and the Shapiro–Wilk test. All analyses were conducted separately for the left and right leg.

Prior to descriptive and statistical analysis, the collected leg volume data were combined with the demographic data of each subject. The demographic data were analyzed for the entire study cohort, and, for both, the complete and limited leg visibility workflow subgroups.

The mean value of paired leg volume measurements from each subject was assessed for the entire study cohort. By splitting the study cohort into five subgroups based on the WHO BMI classification [34], a boxplot was created to graphically analyze the calculated mean volumes in relation to subject BMI. The BMI subgroups were defined by the relevant value in kilogram per square meter (kg/m^2^): normal weight (18.5–24.9), pre-obesity (25.0–29.9), obesity class I (30.0–34.9), obesity class II (35.0–39.9), and obesity class III (above 40).

The difference between the paired absolute leg volume measurements from each subject was analyzed for the entire study cohort as well as for both the complete and limited leg visibility workflow subgroups. Using the paired *t*-test to compare the volume samples, we calculated the mean differences between the samples with 95% confidence intervals.

Furthermore, the relative differences between each pair of repeated measurements were calculated as a percentage of the absolute difference between paired leg volumes divided by the mean value of both measurements. By calculating these relative differences, we could compare the repeated measurements regardless of the varying absolute leg volumes of each subject. We deemed lower values of relative difference between repeated leg volume measurements to indicate an increasing level of precision.

A boxplot was created to graphically analyze the relative difference between each pair of repeated measurements depending on the respective use of workflow. The Wilcoxon test was used to test for statistical significance between the scores of both workflow samples for each leg.

## Results

### Workflow Application

**Fig. 3:**
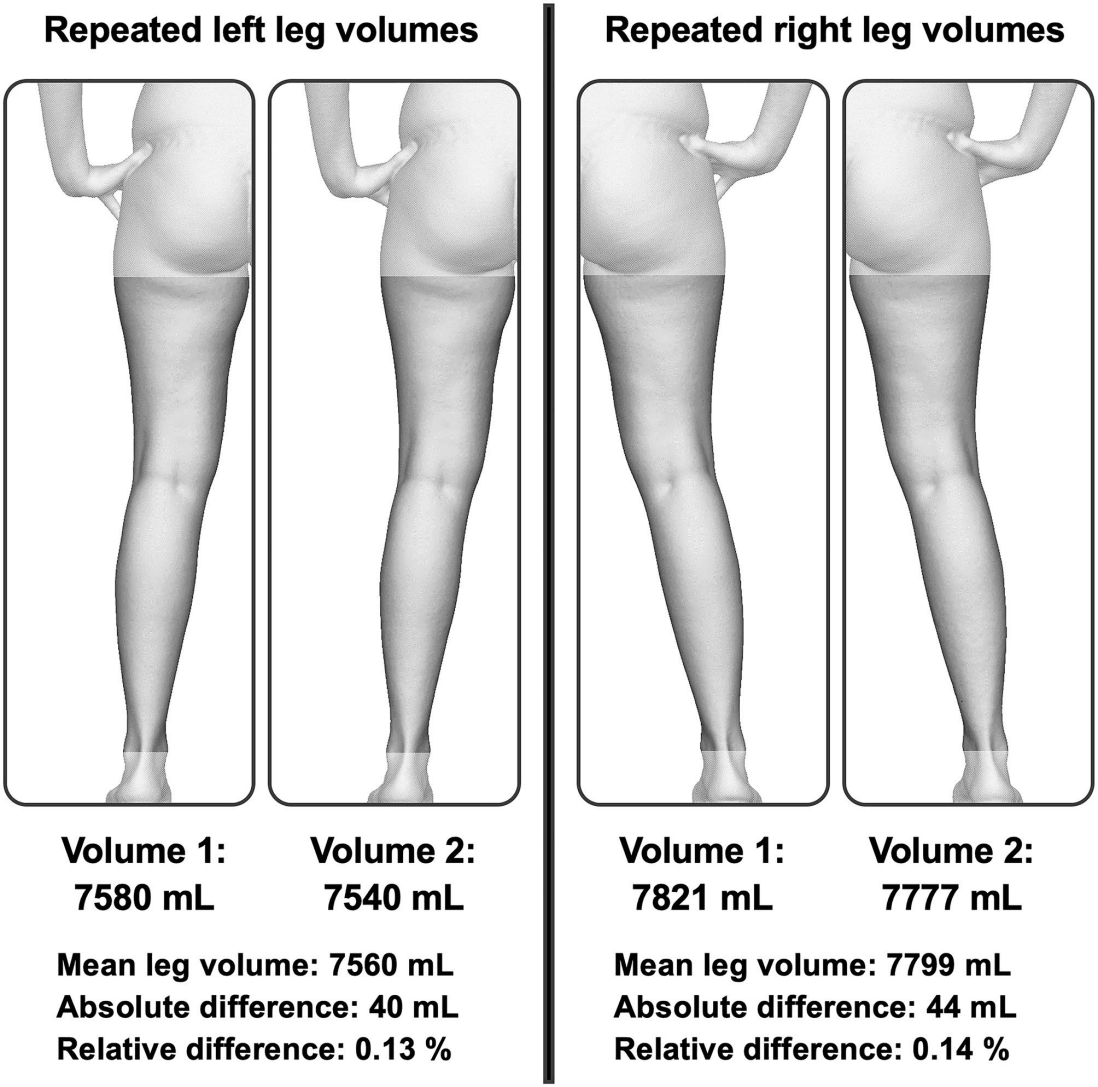
Individual and mean leg volume assessment and calculation of absolute and relative differences between repeated leg scans of paired whole-body scans processed using the complete leg visibility workflow variation

By applying the described workflow, paired sets of whole-body 3D surface data were successfully processed and analyzed for each of the 82 study participants. As illustrated for a single subject in Fig. 3, the two sets of volumetric data for each leg were compared. The respective mean values as well as the absolute and relative differences between repeated leg scans were calculated to gauge workflow precision. Based on the leg morphology within the 3D surface data of the subjects enrolled in this study, 36 subjects were included in the complete leg visibility workflow subgroup and 46 in the limited leg visibility workflow subgroup. A detailed overview regarding the demographic results is listed in Table 1.

**Table 1:** Demographic results

Cohort	Sex	Age	BMI
Entire study cohort	64 female, 18 male	41 ± 12 (21 to 71)	32.3 ± 9.7 (18.7 to 55.8)
Complete leg visibility workflow	27 female, 9 male	38 ± 13 (21 to 71)	26.4 ± 6.9 (18.7 to 44.6)
Limited leg visibility workflow	37 female, 9 male	44 ± 11 (22 to 65)	36.9 ± 9.2 (21.1 to 55.8)

All results are given as mean ± SD (standard deviation) with range in years for age and kilogram per square meter (kg/m^2^) for BMI

### Absolute Leg Volume Measurements

**Fig. 4:**
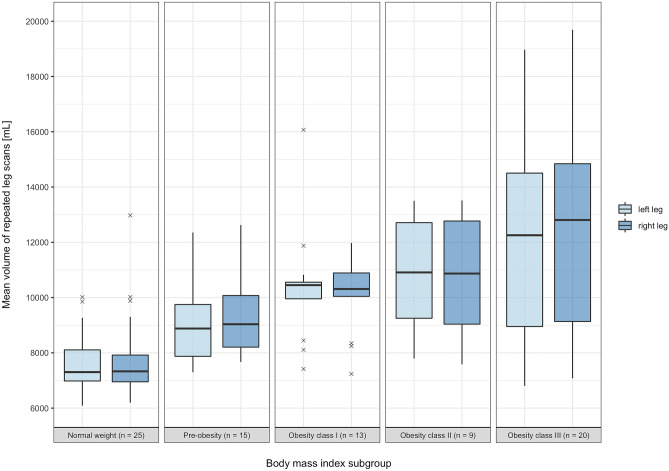
Boxplot depicting the mean repeated left and right leg volume measurements in relation to the defined BMI subgroups

The average of the mean repeated leg volumes of the entire study cohort was 9752 ± 2784 mL (range 6088 to 18,970 mL) for the left side and 9841 ± 2759 mL (range 6194 to 19,690 mL) for the right side. A graphical analysis of the mean bilateral leg volume measurements in relation to the defined BMI subgroups can be seen in Fig. 4. In this graph, there are two outliers of unilateral leg volume without a corresponding value for the other leg. One is in the normal weight BMI subgroup at 12,975 mL (right leg) while the other is in the obesity class I subgroup at 16,070 mL (left leg).

A detailed overview regarding the repeated volume measurement samples as well as the mean differences and 95% confidence intervals is listed in Table 2.

**Table 2:** Leg volume analysis of repeated whole body surface images

Cohort	Leg	Leg volume 1	Leg volume 2	Mean dif	95% CI
Entire study cohort	Left leg	9746 ± 2782	9757 ± 2787	−11.3	−30.9 to 8.3
Entire study cohort	Right leg	9845 ± 2757	9837 ± 2762	7.9	−13.1 to 28.9
Complete leg visibility workflow	Left leg	7856 ± 1148	7857 ± 1150	−0.6	−29.2 to 28.0
Complete leg visibility workflow	Right leg	8015 ± 1212	7993 ± 1227	21.8	−6.2 to 49.9
Limited leg visibility workflow	Left leg	11,225 ± 2794	11,245 ± 2792	−19.6	−47.2 to 8.0
Limited leg visibility workflow	Right leg	11,277 ± 2787	11,280 ± 2778	−3.0	−33.9 to 27.8

All results are given as mean ± SD (standard deviation) in milliliters (mL)

When examining the entire study cohort, there was no statistically significant difference in the scores of the left leg, *t*(81) =  −1.143, *p* = 0.256, and no statistically significant difference in the scores of the right leg, *t*(81) = 0.747, *p* = 0.457.

Furthermore, the analysis for the complete leg visibility workflow subgroup showed no statistically significant difference in the scores of the left leg, *t*(35) =  −0.043, *p* = 0.966, and no statistically significant difference in the scores of the right leg, *t*(35) = 1.581, *p* = 0.123.

The assessment of the limited leg visibility workflow subgroup showed no statistically significant difference in the scores of the left leg, *t*(45) =  −1.433, *p* = 0.159, and no statistically significant difference in the scores of the right leg, *t*(45) =  −0.199, *p* = 0.843.

### Relative Difference Between Repeated Leg Volume Measurements

**Fig. 5:**
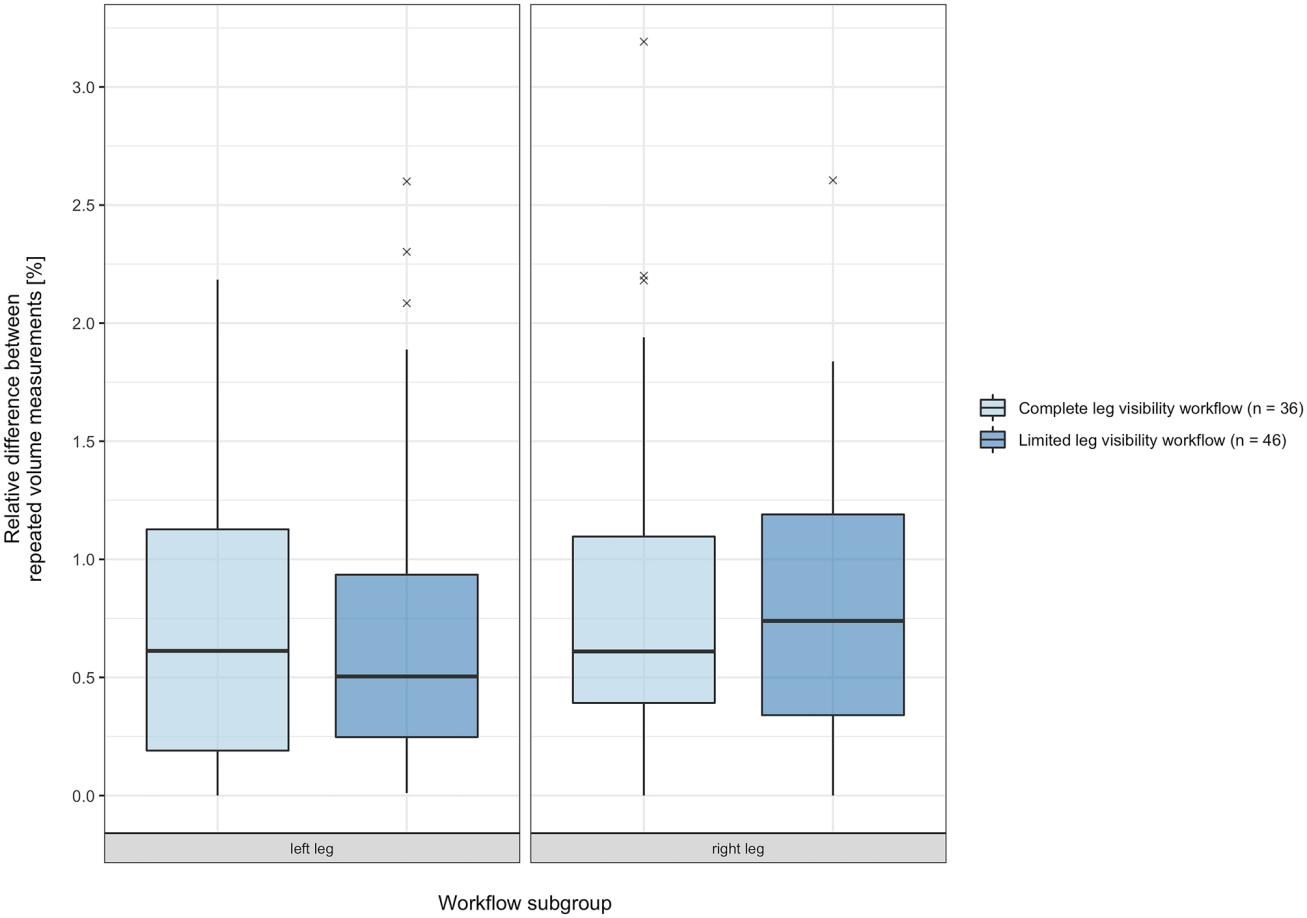
Boxplot depicting the relative differences between repeated left and right leg volume measurements acquired using the complete or limited leg visibility workflow variation

The mean value of relative differences between the repeated leg volumes of the entire study cohort was 0.73 ± 0.62% (range 0.00 to 2.60%) for the left side and 0.82 ± 0.65% (range 0.00 to 3.19%) for right side. A graphical analysis of the samples of relative difference between repeated left and right leg volume measurements acquired using the complete or limited leg visibility workflow can be seen in Fig. 5.

When comparing the samples of relative difference between repeated left leg volume measurements acquired using the complete or limited leg visibility, there was no statistically significant difference in the scores; *W* = 875, *p* = 0.666. The estimated difference in location of the medians was 0.056 (95% confidence interval −0.151 to 0.328).

When comparing the samples of relative difference between repeated right leg volume measurements acquired using either the complete or the limited leg visibility workflow variation, there was no statistically significant difference in the scores, *W* = 836.5, *p* = 0.940. The estimated difference in location of the medians was 0.009 (95% confidence interval 0.268 to 0.263).

## Discussion

In this study, a novel workflow for the measurement of bilateral leg volume from whole-body 3DSI was described and assessed. The data-processing procedure was specifically designed to be applicable not only when the predefined leg positioning allows full visibility of each leg, but also in cases where subject leg morphology does not allow for complete circumferential surface scan of both legs.

**Fig. 6:**
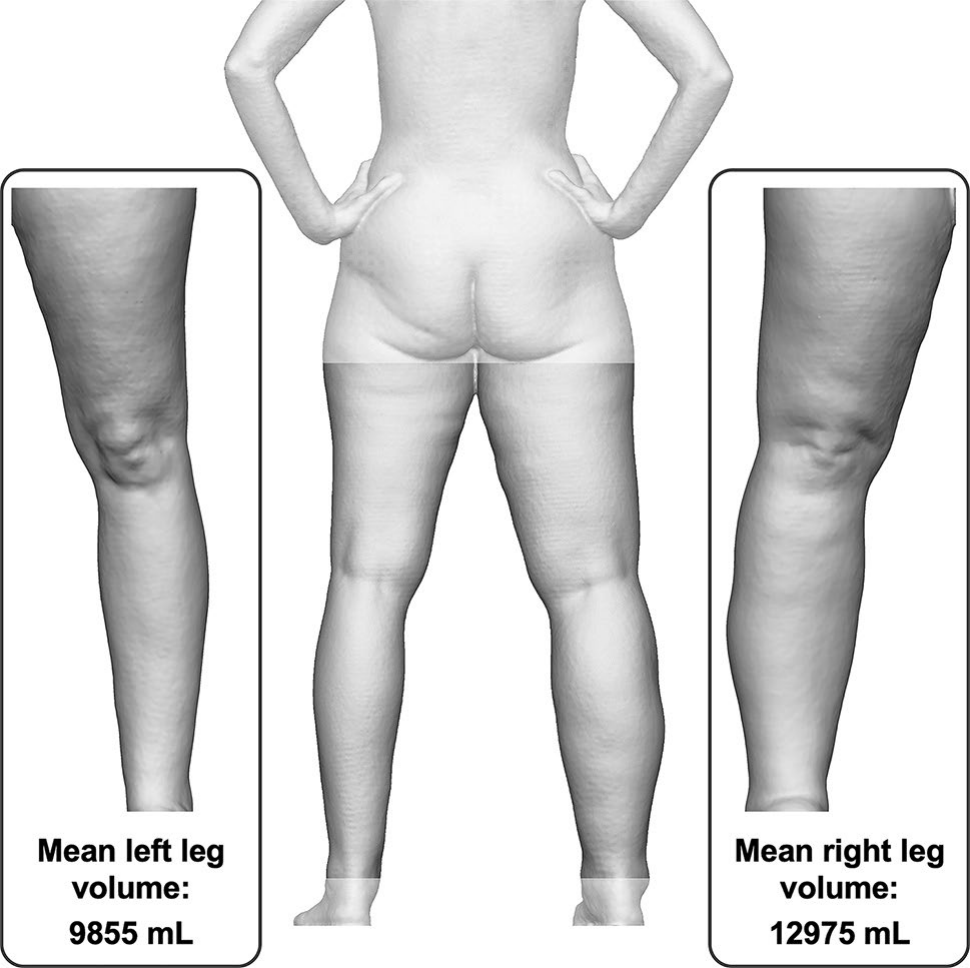
Comparison of the mean left and right leg volume of a 54-year-old female subject with unilateral right-sided lymphedema whose whole-body scans were processed using the limited leg visibility workflow variation

Following the described method, a mobile 3D imaging system consisting of a structured light scanner and an automated turntable was used for paired scans of each of the 82 subjects included in this study. As intended during study enrollment, the cohort consisted of participants with a wide BMI and leg volume range. To examine the relationship between these variables, the mean values of paired repeated leg volumes were graphically assessed dependent on the subject BMI subgroup, as is depicted in Fig. 4. While it is apparent that the subjects included in the obesity class III subgroup had the widest range of leg volume for both legs, there were some subjects with the highest BMI values who nevertheless had similarly low leg volumes to those in the normal BMI subgroup. This may be explained by the body composition of the respective subjects, as some participants had comparatively slim legs despite a bulky torso responsible for the high subject BMI value. When further examining the boxplot, it is of note that there were some outliers of mean leg volume in the normal or obesity I BMI subgroup that were only observed in one leg. The two subjects in question presenting themselves with a respective mean right leg volume of 12,975 mL and mean left leg volume of 16,070 mL were patients suffering from unilateral lymphedema. The normal BMI subgroup subject is depicted in Fig. 6. This observation positively highlights the fact that the workflow can meaningfully distinguish asymmetries, as is highly relevant for future clinical application of the presented workflow.

Because of the increasing need for objective and preferably digital forms of documentation, gathering precise data regarding the human body is growing ever more important in the context of evidence based medicine. Thus, a precision assessment of a novel workflow is an essential step when the new method is intended to be applied in a clinical setting. It is therefore the subject of various 3DSI validation studies [16, 35–40]. In this study, precision was examined by bilaterally comparing the individual volume results of both repeated whole-body scans and by calculating the absolute differences as well as the relative percent difference. We found no statistically significant difference between the paired samples of absolute leg volume results. The estimated absolute differences were low, and by examining the 95 confidence intervals, these small values were further indicative that the differences in leg volume between repeated whole-body scans were of minimal clinical significance. When examining the relative percent differences to assess the deviation between repeated measures regardless of the varying individual leg volumes, we found that the mean values of both legs were below 1%. There were no values above 3.2%. When interpreting these values in a clinical context, it is important to look at the initially calculated absolute leg volumes, e.g., a 1% deviation with a mean volume of 9000 mL would result in a deviation of 90 mL. While not perfect, we deemed these absolute and relative differences between repeated scans to be negligible. We thus concluded that the workflow yielded precise results. When further comparing the relative differences of the complete and limited surface visibility cohorts, we found no statistically significant differences for either leg. We also found similarly low values for both workflow subgroups. This is depicted in Fig. 5. We therefore assumed that both worfklow variations were of similarly high precision. We thus concluded that the use of the estimation algorithm applied in the limited leg visibility workflow yields comparable results to the volume measurement conducted on completely circumferentially visible legs. We deemed these results indicative of the usefulness of standardized whole-body 3DSI as a tool to gauge changes in leg volume in clinical practice.

Nonetheless, the use of whole-body 3DSI is associated with certain drawbacks. Subjects are required to stand in an upright position with the body weight borne by both legs. This method is therefore of limited use for subjects receiving reconstructive limb surgery or in the case of limb amputation. Methods that individually assess each leg may be more useful in such cases, such as CT, MRI, or a single-leg 3DSI-based approach [12]. Also, our scanning process using a handheld scanner takes up to a minute. This requires subjects to remain standing in a fixed pose on a revolving turntable for the scan duration. In contrast, a permanently installed stereo-photogrammetric system is capable of near-instantaneous scanning. The 3DSI system presented in this study has the benefit of being a compact and portable setup and can thus allow bedside imaging. However, it may not be suitable for subjects who are prone to dizziness or with an impaired sense of balance. While the scanning duration and use of a turntable may raise the question of movement artifacts, the software used for data-processing implements a registration algorithm to align the individual scan frames. Together with the subject positioning and the instruction that patients keep their eyes open, a minimal number of movement artifacts were observed throughout the image acquisition for our study population. These artefacts were found in the upper extremity or for the head, but not for the lower extremity. We thus concluded our scanning workflow to be a robust tool for volumetric leg assessment.

A strength of this study lies in the relatively large sample size and morphologic variety of the examined subjects. While some studies assessing the precision of a workflow have examined rather small and homogenous cohorts or make use of an imaging phantom [16, 26, 39], this study included over 80 subjects in its analysis. A high number of subjects were subsequently available for each of the subgroups that were further analyzed as part of this study. Also, this study specifically examined subjects in the target population for clinical leg volume documentation. As such, it included patients with varying degrees of uni- or bilaterally high values of leg volume, and with a range of BMI values from 18.7 to 55.8 kg/m^2^. While the study investigates measurement reproducibility between two separate scans in a single session, it does not provide information regarding the precision when more than two scans are conducted in one session, or when the scans are spread out over an extended period of time. Also, this study did not include multiple assessments of the same subjects by different assessors, and thus, inter-rater reliability was not examined. Future studies ought to evaluate reproducibility by repeatedly examining the same cohort without the subjects undergoing pronounced morphologic changes in the meantime. The influence of varying imaging operators should also be investigated. Finally, future efforts should be made to automate the data-processing part of the workflow.

## Conclusions

The workflow presented in this study to gauge leg volume from whole-body 3D surface data was successfully applied in 82 cases. Both the standard and limited leg visibility workflow variations yielded results for leg volume quantification that were similarly precise. The workflow thus provides a precise method to digitally monitor leg volume regardless of leg morphology and could aid in objectively comparing medical treatment options of the leg in a clinical setting. Whole-body scans acquired using the described 3DSI routine may allow simultaneous assessment of other changes in body morphology after further validation.

## References

[1] Kroh A, Peters F, Alizai PH, Schmitz S, Hölzle F, Neumann UP, Ulmer FT, Modabber A (2020). 3D optical imaging as a new tool for the objective evaluation of body shape changes after bariatric surgery. Obes. Surg..

[2] Ritschl LM, Wolff KD, Erben P, Grill FD (2019). Simultaneous, radiation-free registration of the dentoalveolar position and the face by combining 3D photography with a portable scanner and impression-taking. Head Face Med..

[3] Sutherland J, Belec J, Sheikh A, Chepelev L, Althobaity W, Chow BJW, Mitsouras D, Christensen A, Rybicki FJ, La Russa DJ (2019). Applying modern virtual and augmented reality technologies to medical images and models. J. Digit. Imaging..

[4] Wilting FNH, Hameeteman M, Tielemans HJP, Ulrich DJO, Hummelink S (2020). Three-dimensional evaluation of breast volume changes following autologous free flap breast reconstruction over six months. Breast..

[5] Koban KC, Etzel L, Li Z, Pazos M, Schönecker S, Belka C, Giunta RE, Schenck TL, Corradini S (2020). Three-dimensional surface imaging in breast cancer: a new tool for clinical studies?. Radiat. Oncol..

[6] Cotofana S, Koban KC, Konstantin F, Green JB, Etzel L, Giunta RE, Schenck TL (2019). The surface-volume coefficient of the superficial and deep facial fat compartments: a cadaveric three-dimensional volumetric analysis. Plast. Reconstr. Surg..

[7] Speir EJ, Matthew Hawkins C, Weiler MJ, Briones M, Swerdlin R, Park S, Brandon Dixon J (2019). Volumetric assessment of pediatric vascular malformations using a rapid, hand-held three-dimensional imaging system. J. Digit. Imaging..

[8] Cau, N., Corna, S.: Circumferential versus hand-held laser scanner method for the evaluation of lower limb volumes in normal-weight and obese subjects. J. Nov. Physiother. 6, (2016). 10.4172/2165-7025.1000303

[9] Tokumoto H, Akita S, Kuriyama M, Mitsukawa N (2018). Utilization of three-dimensional photography (VECTRA) for the evaluation of lower limb lymphedema in patients following lymphovenous anastomosis. Lymphat. Res. Biol..

[10] Chromy, A., Zalud, L., Dobsak, P., Suskevic, I., Mrkvicova, V.: Limb volume measurements: comparison of accuracy and decisive parameters of the most used present methods. Springerplus. 4, (2015). 10.1186/s40064-015-1468-7 10.1186/s40064-015-1468-7 PMC4653131 26618096

[11] Tan CW, Coutts F, Bulley C (2013). Measurement of lower limb volume: agreement between the vertically oriented perometer and a tape measure method. Physiotherapy..

[12] Mestre S, Veye F, Perez-Martin A, Behar T, Triboulet J, Berron N, Demattei C, Quéré I (2014). Validation of lower limb segmental volumetry with hand-held, self-positioning three-dimensional laser scanner against water displacement. J. Vasc. Surg. Venous Lymphat. Disord..

[13] Koban KC, Titze V, Etzel L, Frank K, Schenck T, Giunta R (2018). Quantitative volumetrische Analyse der unteren Extremität: Validierung gegenüber etablierter Maßbandmessung und Wasserverdrängung. Handchir Mikrochir Plast Chir..

[14] Meijer RS, Rietman JS, Geertzen JH, Bosmans JC, Dijkstra PU (2004). Validity and intra- and interobserver reliability of an indirect volume measurements in patients with upper extremity lymphedema. Lymphology..

[15] Bolt A, De Boer-Wilzing VG, Geertzen JHB, Emmelot CH, Baars ECT, Dijkstra PU (2010). Variation in measurements of transtibial stump model volume: a comparison of five methods. Am. J. Phys. Med. Rehabil..

[16] Seminati, E., Canepa Talamas, D., Young, M., Twiste, M., Dhokia, V., Bilzon, J.L.J.: Validity and reliability of a novel 3D scanner for assessment of the shape and volume of amputees’ residual limb models. PLoS One. 12, e0184498 (2017). 10.1371/journal.pone.0184498 10.1371/journal.pone.0184498 PMC5590959 28886154

[17] Dessery, Y., Pallari, J.: Measurements agreement between low-cost and high-level handheld 3D scanners to scan the knee for designing a 3D printed knee brace. PLoS One. 13, (2018). 10.1371/journal.pone.0190585 10.1371/journal.pone.0190585 PMC5761889 29320560

[18] Lin JD, Chiou WK, Weng HF, Tsai YH, Liu TH (2002). Comparison of three-dimensional anthropometric body surface scanning to waist-hip ratio and body mass index in correlation with metabolic risk factors. J. Clin. Epidemiol..

[19] Ng, B.K., Hinton, B.J., Fan, B., Kanaya, A.M., Shepherd, J.A.: Clinical anthropometrics and body composition from 3D whole-body surface scans. Eur. J. Clin. Nutr. Adv. online Publ. (2016). 10.1038/ejcn.2016.109 10.1038/ejcn.2016.109 PMC5466169 27329614

[20] Wang J, Gallagher D, Thornton JC, Yu W, Horlick M, Pi-Sunyer FX (2006). Validation of a 3-dimensional photonic scanner for the measurement of body volumes, dimensions, and percentage body fat. Am. J. Clin. Nutr..

[21] Lu JM, Wang MJJ (2008). Automated anthropometric data collection using 3D whole body scanners. Expert Syst. Appl..

[22] Schwarz-Müller F, Marshall R, Summerskill S (2018). Development of a positioning aid to reduce postural variability and errors in 3D whole body scan measurements. Appl. Ergon..

[23] Rapprich S, Dingler A, Podda M (2011). Liposuction is an effective treatment for lipedema - results of a study with 25 patients. JDDG - J. Ger. Soc. Dermatology..

[24] Norton J, Donaldson N, Dekker L (2002). 3D whole body scanning to determine mass properties of legs. J. Biomech..

[25] Chiu CY, Pease DL, Fawkner S, Sanders RH (2018). Automated body volume acquisitions from 3D structured-light scanning. Comput. Biol. Med..

[26] Etzel L, Koban KC, Li Z, Frank K, Giunta RE, Schenck TL (2019). Whole Body Surface Assessment – Implementierung und Erfahrungen von 360° 3D Ganzkörperscans: Möglichkeiten zur Objektivierung und Verlaufskontrolle an den Extremitäten und am Körperstamm. Handchir Mikrochir Plast Chir..

[27] R Core Team: R: a language and environment for statistical computing. (2020)

[28] RStudio Team: RStudio: integrated development environment for R. (2020)

[29] Rmarkdown: Dynamic documents for R. (2020)

[30] Wickham, H., Averick, M., Bryan, J., Chang, W., McGowan, L.D., François, R., Grolemund, G., Hayes, A., Henry, L., Hester, J., Kuhn, M., Pedersen, T.L., Miller, E., Bache, S.M., Müller, K., Ooms, J., Robinson, D., Seidel, D.P., Spinu, V., Takahashi, K., Vaughan, D., Wilke, C., Woo, K., Yutani, H.: Welcome to the tidyverse. Journal of Open Source Software. 4, 1686 (2019). 10.21105/joss.01686

[31] Bache, S.M., Wickham, H.: Magrittr: a forward-pipe operator for R. (2014)

[32] Gohel, D.: Officer: Manipulation of Microsoft Word and Powerpoint documents. (2020)

[33] Gohel, D.: Flextable: functions for tabular reporting. (2020)

[34] World Health Organization: body mass index - BMI (accessed 2021–05–30), https://www.euro.who.int/en/health-topics/disease-prevention/nutrition/a-healthy-lifestyle/body-mass-index-bmi

[35] Anik AA, Xavier BA, Hansmann J, Ansong E, Chen J, Zhao L, Michals E (2020). Accuracy and reproducibility of linear and angular measurements in virtual reality: a validation study. J. Digit. Imaging..

[36] Jeon JH, Kim DY, Lee JJ, Kim JH, Kim WC (2016). Repeatability and reproducibility of individual abutment impression, assessed with a blue light scanner. J. Adv. Prosthodont..

[37] Eder M, Brockmann G, Zimmermann A, Papadopoulos MA, Schwenzer-Zimmerer K, Zeilhofer HF, Sader R, Papadopulos NA, Kovacs L (2013). Evaluation of precision and accuracy assessment of different 3-D surface imaging systems for biomedical purposes. J. Digit. Imaging..

[38] Kovacs L, Zimmermann A, Brockmann G, Baurecht H, Schwenzer-Zimmerer K, Papadopulos NA, Papadopoulos MA, Sader R, Biemer E, Zeilhofer HF (2006). Accuracy and precision of the three-dimensional assessment of the facial surface using a 3-D laser scanner. IEEE Trans. Med. Imaging..

[39] de Menezes M, Rosati R, Ferrario VF, Sforza C (2010). Accuracy and reproducibility of a 3-dimensional stereophotogrammetric imaging system. J. Oral Maxillofac. Surg..

[40] Adler C, Steinbrecher A, Jaeschke L, Mähler A, Boschmann M, Jeran S, Pischon T (2017). Validity and reliability of total body volume and relative body fat mass from a 3-dimensional photonic body surface scanner. PLoS One..

